# Neurosurgical application of olaparib from a thermo-responsive paste potentiates DNA damage to prolong survival in malignant glioma

**DOI:** 10.1038/s41416-024-02878-2

**Published:** 2024-10-22

**Authors:** Riccardo Serra, Stuart J. Smith, Jonathan Rowlinson, Noah Gorelick, Cara Moloney, Phoebe McCrorie, Gareth J. Veal, Philip Berry, Anthony J. Chalmers, Ian Suk, Kevin M. Shakesheff, Cameron Alexander, Richard G. Grundy, Henry Brem, Betty M. Tyler, Ruman Rahman

**Affiliations:** 1 Department of Neurosurgery, Johns Hopkins University, Baltimore, USA; 2 Children’s Brain Tumour Research Centre, School of Medicine, Biodiscovery Institute, University of Nottingham, Nottingham, UK; 3 Newcastle University Centre for Cancer, Newcastle University, Newcastle, UK; 4 School of Cancer Sciences, University of Glasgow, Glasgow, UK; 5 The Open University, Milton Keynes, UK; 6 School of Pharmacy, University of Nottingham, Nottingham, UK; 7 Departments of Biomedical Engineering, Oncology and Ophthalmology, Johns Hopkins University, Baltimore, USA

**Keywords:** CNS cancer, Oncology

## Abstract

**Background:**

There is increased pan-cancer specific interest in repurposing the poly adenosine diphosphate-ribose polymerase-1 (PARP-1) inhibitor, olaparib, for newly diagnosed or recurrent isocitrate dehydrogenase wild type glioblastoma. We explore whether intra-cavity delivery of olaparib confers a survival benefit in a pre-clinical high-grade glioma model.

**Methods:**

Primary tumor RNA sequencing data was used to determine PARP-1 as a target in the glioblastoma infiltrative margin. We assessed radiosensitization conferred by olaparib alone and concomitant to genotoxic insults in vitro using clonal growth assays, cell cycle analysis and immunocytochemistry, and in vivo upon post-surgical delivery from a temperature-sensitive polymeric paste.

**Results:**

RNA-sequencing confirmed PARP-1 as a viable therapy target in glioblastoma infiltrative disease. Acute exposure of glioma cells to olaparib impaired proliferation and induced late-stage apoptosis associated with DNA damage in vitro, potentiated by radiation. Using high-grade glioma orthotopic allografts, a long-term overall survival benefit was observed upon interstitial olaparib delivery concomitant with radiotherapy, compared to systemic olaparib and standard glioblastoma treatment. Combined delivery of olaparib with either temozolomide or etoposide increased long-term survival, suggestive of olaparib functioning as DNA damage sensitizer.

**Conclusions:**

Collectively, our data support a rationale for localized olaparib delivery concomitant with the current clinical regimen for malignant glioma treatment.

## Background

The World Health Organization classified grade 4 gliomas represent the most malignant and genetically heterogeneous group of de novo brain tumors. The most common sub-type is grade 4 isocitrate dehydrogenase wild type (IDH-WT) glioblastoma (GBM) with an age standardized global incidence of 4.6/100,000/year [1]. Despite DNA damaging radio-chemotherapy post-surgery, mean survival rates remain dismal at ~10–15 months from IDH-WT GBM diagnosis. Current 5-year survival rates are 22% (patients aged 20–44), 9% (patients aged 45–54) and 6% (patients aged 55-64), with limited survival increase over several decades [2–4]. Disappointingly, no new pharmacotherapies have been shown to prolong overall survival in a phase III randomized trial to treat IDH-WT GBM since the introduction of temozolomide (TMZ) in 2005 [5], and therefore the promise of efficacious drug repurposing has not been realized clinically. Furthermore, despite surgery remaining frontline therapy for most patients, no post-surgical interstitial drug delivery technologies have been clinically adopted since carmustine-loaded Gliadel® wafers two decades ago [6–12].

The nuclear protein poly (ADP-ribose) polymerase-1 (PARP-1) is well characterized for pleiotropic functions in diverse pathophysiological processes such as DNA repair, transcription, apoptosis, and inflammation [13], and is overexpressed in many cancers [14]. The orally administered bioavailable small molecule inhibitor of PARP-1, olaparib (OLA), is effective as a single agent against tumors characterized by defects in homologous recombination DNA repair [15, 16] and is approved (as Lynparza) by both the Food and Drug Administration and European Medicines Agency for maintenance treatment of BRCA-mutated advanced ovarian cancer [17], HER2-negative high-risk early breast cancer [18], newly diagnosed advanced pancreatic cancer [19], and recurrent prostate cancer [20]. Accordingly, there is considerable current interest in assessing OLA as a sensitizer to the DNA damaging agents routinely used to treat IDH-WT GBM [21–23]. Despite poor intact blood-brain-barrier penetration of OLA in a non-diseased model, a phase I clinical trial (OPARATIC) detected systemically delivered OLA in GBM core and margin regions at ~500 nM concentration, with OLA well tolerated when combined with the standard treatment of TMZ alkylating agent [24]. A phase I/IIa study to evaluate OLA as a radiosensitizer in unresectable or partially resectable GBM is currently ongoing [25]. Furthermore, preclinical data indicates that the radiosensitizing effects of PARP inhibitors such as OLA are dose-dependent [26] and thus it would likely be beneficial to expose tumor cells to higher concentrations of OLA than can be achieved by systemic treatment.

Whilst focused ultrasound-mediated delivery of OLA was recently reported to confer radiosensitization in a diffuse midline glioma model [27], and nanoparticles encapsulated with the PARP inhibitor talazoparib was shown to induce tumor regression and reduce leptomeningeal spread in a preclinical metastatic model of medulloblastoma [28], there have been no reported studies assessing localized OLA delivery in a cortical high grade glioma model. We previously developed an interstitial drug delivery system applied to the tumor resection cavity, consisting of biodegradable polymer microparticles made from blends of poly(DL-lactic*-co-*glycolic acid) (PLGA) and poly(ethylene glycol) (PEG). The PLGA/PEG delivery system is applied intra-operatively as a paste that molds to the resection cavity lining and sinters (through fusing of microparticles through heat and pressure) at body temperature as the microparticles fuse, retaining close apposition to the cavity lining [29]. We reported a significant long-term survival benefit compared to clinical standard treatment, in high-grade glioma rat orthotopic allografts treated with combined TMZ and the topoisomerase II inhibitor, etoposide (ETOP) (not clinically approved for GBM), delivered by PLGA/PEG paste after surgery, an effect further potentiated by concomitant radiotherapy [30]. Moreover, we have recently demonstrated feasibility of local administration of OLA from a drug delivery system and confirmed brain parenchyma OLA penetration ex vivo [31–33]. Here, we sought to complement currently progressing GBM clinical trials of systemic OLA administration, by evaluating intra-cavity delivery of high concentrations of OLA against GBM standard therapies of radiation and TMZ. We test an overarching hypothesis that interstitially delivered OLA-mediated radio- and/or DNA damage-sensitization confers a significant survival benefit compared to standard clinical treatment, and to systemically delivered OLA.

## Methods

### Transcriptomics data analyzes

Using the R2: Genomics Analysis and Visualization Platform, PARP-1 median gene expression (log2) was retrieved from two normal brain cohorts, and three IDH-1 WT primary GBM cohorts (*n* = 20, *n* = 70 and *n* = 74) containing both MGMT methylated and unmethylated tumors.

### Tumor cell culture

U251 (GBM) and 9 L (rat gliosarcoma grade 4) cell lines were propagated in Dulbecco’s Modified Eagle Medium (DMEM) (Sigma-Aldrich) containing 1 g/L glucose, which was supplemented with 10% fetal bovine serum (FBS) (GE Healthcare) and 1% L-glutamine (Sigma-Aldrich). Both cell lines were mycoplasma-free. U251 was STR genotyped (Eurofins) to confirm absence of cross-contamination.

### Clonogenic assay

9 L cells at a seeding density of 5.0 × 10^2^ cells/well, was used to determine clonogenic potential, prior to treatment with either 3 µM OLA, 3 Gy XRT or a combination of OLA/XRT, and incubated for 7 days. Crystal violet (Sigma–Aldrich, St. Louis, MO, USA) was used to stain fixed cells, prior to light microscopy imaging. Data was obtained with three biological replicates per experiment.

### Annexin staining

Flow cytometry was used to measure early- (AnnexinV positive) and late-stage (AnnexinV-Propidium Iodide (PI) positive) apoptotic cells after 96 h from initial treatment, with mean proportion (%) of cells calculated from three independent repeats. 9 L and U251 cells at a seeding density of 5.0 ×10^4^ cells/well, were treated with either 2 µM OLA, 10 Gy XRT, a combination of OLA/XRT, 500 µM TMZ, a combination of OLA/XRT/TMZ, 500 nM ETOP, or a combination of OLA/XRT/ETOP. Cells were enzymatically dissociated using trypsin, washed with PBS, and incubated with AnnexinV Ab and PI (Invitrogen, Carlsbad, CA, USA) prior to analyzes. An untreated control was included for comparison.

### Immunocytochemistry

Cells were assessed by immunofluorescence after 96 h from initial treatment. U251 and 9 L cells were seeded at a density of 1.0 × 10^4^ cells/well, and treated with OLA 2 uM, TMZ 500 uM, ETOP 500 nM, combination OLA/XRT, combination OLA/XRT/TMZ, and combination OLA/XRT/ETOP. To detect nuclear expression of γH2AX, fixed cells were incubated with primary antibody (anti-phospho-Histone H2A.X (Ser139) 1:500, Merk-Millipore, Catalog No. 05-636) 96 h after treatment, and counterstained with a secondary antibody (Jackson Laboratory, Bar Harbor, ME, USA), and DAPI (Invitrogen, Carlsbad, CA, USA). Slides were prepared with mounting medium (Dako, Santa Clara, CA, USA) and imaged with a Zeiss microscope.

### Formulation of PLGA/PEG microparticle matrices

Temperature-sensitive polymer particles were manufactured from blends of 53 kDa P_DL_LGA (85:15 DLG 4CA; Evonik Industries) and PEG400 (Sigma–Aldrich) as described previously [29]. Briefly, a mixture of 93.5%:6.5% PLGA/PEG (w/v) was blended at 80–90 °C using a hotplate. To obtain 100 to 200 mm particle size fractions, cooled polymer was finely ground into particles and sieved.

### Olaparib in vitro release from PLGA/PEG

300 mg of PLGA/PEG microparticles were mixed with 236 µL PBS containing 3 mg of OLA at room temperature. The paste was then applied into six polytetrafluoroethylene cylindrical molds (4 ×6 mm) and incubated for 30 min at 37 °C. In vitro drug release was conducted using six independent repeats (polymer matrix molds). To monitor in vitro release, the six molds were placed in 5 mL of PBS and incubated at 37 °C. At given time intervals, PBS was removed, retained, and replaced with a fresh aliquot. The retained fractions were assayed using HPLC for quantification of OLA release.

### High-performance liquid chromatography

OLA quantification was conducted on an Agilent Technologies 1200 Series HPLC system using an ACE 5 C_18_ column (250 × 4.6 mm plus an ACE 5 C_18_ 10 × 3 mm guard column) maintained at 25 °C. A mobile phase of ammonium acetate (10 mM, pH= 4)/acetonitrile (55/45 v/v) was used at a flow rate of 1 mL/min for 6 min and the eluent was monitored at 254 nm. A standard curve over the range 0.05–50 µg/mL OLA in matched matrix was prepared.

### Animals

F344 immunocompetent female rats (6–7 months old) weighing 160-200 grams were maintained in well-ventilated cages (Harlan Bioproducts) within a barriered unit. All cages were illuminated by fluorescent lights set to a 12-hour light-dark cycle (7am–7pm), as per U.S. Public Health Service Policy on Humane Care and Use of Laboratory Animals guidelines. All animals were treated in accordance with the policies and guidelines of the Johns Hopkins University Animal Care and Use Committee.

### Orthotopic allografts

9 L high-grade glioma tumor was first surgically excised from subcutaneous flanks of carrier animals after humane sacrifice (intraperitoneal overdose of 200 mg/kg sodium pentobarbital) and sliced into 2mm^3^ allografts. Prior to intracranial implants, rats were anesthetized with an intraperitoneal injection of 3 mL/kg of a stock solution containing ketamine hydrochloride, 75 mg/mL (Ketathesia, Butler Animal Health Supply), 7.5 mg/mL xylazine (Lloyd Laboratories) and 14.25% ethyl alcohol in 0.9% NaCl. Ethanol and prepodyne was applied to the shaved surgical area and a burr-hole 3 mm in diameter placed in the left parietal bone after midline scalp incision, with its center 5 mm posterior and 3 mm lateral to bregma. A 2 mm^3^ allograft was then placed in a small region of resected cortex following incision through the dura and cortex, and the wound sealed using sterile autoclips. After 5 days to permit tumor uptake and growth, the incision was re-opened, and a fine suction tip and biopsy punch used to surgically resect tumor to the tumor–parenchyma interface, thereby mimicking the GBM clinical scenario.

### In vivo efficacy

For in vivo studies, animals were weighed daily to infer appetite loss, and overall survival of treatment versus control arms analyzed using the Kaplan–Meier estimator. **Randomization**: Animals were assigned randomly (coin-flip) to treatment or control arms (1:1 ratio) for the efficacy study, and data collected per arm. **Blinding**: The allocation of animal groups was blinded to care staff where possible, to ensure animal husbandry was invariable for all experimental animals. **Sample size**: A sample size of *n* = 7 per treatment arm was determined by the Wilcoxon–Mann–Whitney test as appropriate for in vivo efficacy assessment. This was based on 80% power (5% significance; two-sided difference of means), where a standardized effect size (signal/noise ratio of 1.6) was estimated from our previous tolerability studies comparing each individual treatment arm for localized delivery of TMZ and ETOP, versus surgery only control [30]. **Rules for stopping data collection**: Animal weight loss/food avoidance and/or neurological deficit were deemed end-points due to treatment-related adverse effects, at which stage, animals were humanely euthanized. **Data inclusion/exclusion criteria**: Inclusion criterion for the in vivo study was an animal weight range of 160-200 grams. Exclusion criterion was adverse complications due to surgery. **Selection of endpoints**: Long-term survivorship was deemed 120 days post-treatment. Oral TMZ was administered to animals at 50 mg/kg/day for 5 days (days 5–9) and XRT administered as an external beam single dose of 10 Gy immediately after surgery, to mimic the full complement of GBM therapy [2]. Rats were randomized into one of the following control or treatment arms with *n* = 7 per arms: (*Control arms*) surgery alone; surgery + intraperitoneal OLA (50 mg/kg daily for 5 days) + XRT; surgery + XRT + oral TMZ by gavage (clinical standard-of-care); (*Treatment arms*) surgery + PLGA/PEG loaded with 10% *w/w* OLA; surgery + PLGA/PEG loaded with 20% *w/w* OLA; surgery + PLGA/PEG loaded with 10% *w/w* OLA + XRT; surgery + PLGA/PEG loaded with 20% *w/w* OLA + XRT; surgery + PLGA/PEG loaded with 10% *w/w* OLA / 20% *w/w* TMZ + XRT; surgery + PLGA/PEG loaded with 20% *w/w* OLA / 20% *w/w* TMZ + XRT; surgery + PLGA/PEG loaded with 10% *w/w* OLA / 50% *w/w* ETOP + XRT; surgery + PLGA/PEG loaded with 20% *w/w* OLA / 50% *w/w* ETOP + XRT. Animals were evaluated post-operatively daily for up to 120 days (whereby LTS was established) and monitored for signs of appetite loss and neurological deficit. Overall survival was determined, animals euthanized, and post-sacrificial brains stored in formalin for histological analyzes.

### Histological analyzes

Thin tissue sections were prepared from fixed (4% paraformaldehyde) whole rat brains. The surgical resection boundary was first identified, and a series of 5 µm sections cut proximal from this site. After overnight incubation at 37 °C, xylene was used to deparaffinize slides, prior to hydration washes using ethanol at decreasing concentrations. Harris hematoxylin (Surgipath, UK) was used for counterstaining and slides which were finally dehydrated and mounted on a slide scanner for microscopy.

### Statistical analyzes

Statistical analyzes were performed using GraphPad Prism Software (Version 9.0, GraphPad Software, San Diego, CA, USA). One-way ANOVA was used to compare PARP-1 gene expression across normal brain and GBM transcriptomic datasets, and between GBM intratumor regions, including 5ALA/FACS positive and negative subpopulations. For survival studies, survival was analyzed using the Kaplan–Meier estimator. Each group was compared to the control groups, and to each of the other treatment arms to study the effect of each single treatment and therapy combinations. Ninety-five confidence intervals were chosen, and asymmetrical confidence intervals were included. Multiple comparisons were performed, and statistical significance was established using Log-rank (Mantel-Cox) test analysis. *p*-values of <0.05 were chosen for significance.

## Results

### Olaparib target gene expression in primary GBM

**Fig. 1:**
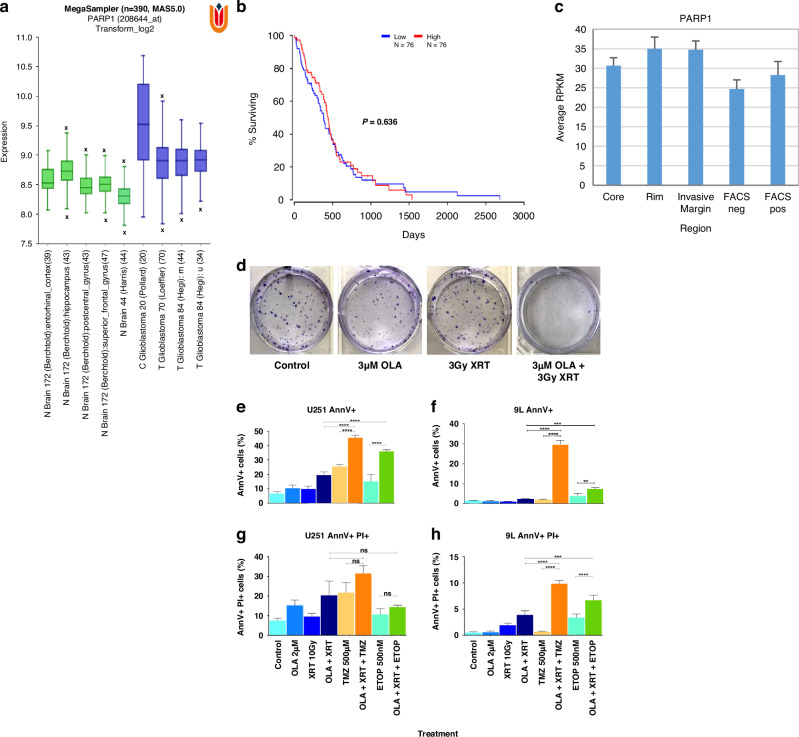
PARP-1 inhibition is a clinically relevant target in primary GBM and confers high-grade glioma sensitivity to DNA damage. **a** Using the R2: Genomics Analysis and Visualization Platform, PARP-1 median gene expression (log2) was retrieved from two normal brain cohorts (*n* = 172 and *n* = 44; green bar plots), and three IDH-1 WT GBM cohorts (*n* = 20, *n* = 70 and *n* = 74; blue bar plots and where ‘u’ and ‘m’ designates unmethylated and methylated MGMT promoter respectively). One-way ANOVA (F statistic – 27.83; *p*-value < 0.01) indicates variance in PARP-1 expression across groups, with higher expression in primary GBM, relative to normal brain (median gene expression range of 8.31-8.73 for normal brain regions, 8.91–9.52 for IDH-1 WT GBM). **b** Kaplan–Meier survival analyzes on TCGA (*n* = 76) IDH-1 WT GBM, based upon PARP-1 gene expression, showing no significant difference between high and low expression cohorts (log rank *p*-value 0.636). **c** PARP-1 gene expression (reads per kilobase million; RPKM) respectively across intra-tumor GBM biopsies (core, peripheral rim, unsorted invasive margin) and fluorescence-activated cell sorted invasive margin tumor (FACS pos.) and non-tumor (FACS neg.) based upon 5ALA fluorescence. No significant difference in PARP-1 mean expression was observed between the clinically relevant FACS/5ALA positive invasive GBM sub-population(s), and all intra-tumor regions and FACS/5ALA negative sub-population(s) (One-way ANOVA *p*-value 0.58, *f*-ratio 2.44). **d** 9 L clonogenic potential is significantly reduced after 7 days of exposure to a combination of 3 µM OLA and 3 Gy XRT, relative to control or each treatment alone. **e**–**h** The proportion of single-positive (AnnV^+^-PI^-^) and double-positive (AnnV^+^-PI^+^) cells significantly increased when either U251 human GBM or 9 L rat gliosarcoma cells were exposed to a combination of OLA/XRT/TMZ and OLA/XRT/ETOP, relative to vehicle-only control, each treatment alone, or a combination of OLA/XRT (****p < 0.0001; ***p = 0.0001, **p = 0.001, *p = 0.01 (with the exce*p*tion of a non-significant difference between OLA/XRT/ETOP vs. OLA/XRT or ETOP alone in U251 cells for the AnnV^+^-PI+ fraction).

At the outset, to clinically validate PARP-1 as a viable therapeutic target for IDH-1 WT GBM, we compared gene expression of PARP-1 across publicly accessible transcriptomic datasets. PARP-1 median gene expression (log2) was retrieved from two normal brain cohorts (*n* = 172 and *n* = 44) and three IDH-1 WT GBM cohorts (*n* = 20, *n* = 70 and *n* = 74) including two stratified by O^6^-methylguanine-DNA-methyltransferase (MGMT) promoter methylation status where available. PARP-1 expression was significantly higher (*p* < 0.01) in primary GBM, relative to normal brain (Fig. 1a). Kaplan–Meier survival analyzes on The Cancer Genome Atlas (TCGA) IDH-1 WT GBM (*n* = 76) based upon PARP-1 gene expression revealed no significant difference between high- and low-expression cohorts (log rank *p*-value 0.636) (Fig. 1b).

Next, we sought to specifically ensure OLA-mediated PARP-1 inhibition represents a clinically accurate treatment strategy for localized, interstitial delivery targeting post-surgical residual GBM. We previously reported distinct transcriptomic profiles (RNAseq) of primary GBM infiltrative margin relative to intratumor regions based on 5-aminolevulinic acid (5ALA)-mediated fluorescence sorting [34, 35]. We retrieved this dataset and confirmed no significant difference in PARP-1 mean gene expression between the clinically relevant 5ALA positive invasive GBM sub-population(s), and all intra-tumor regions and 5ALA negative sub-population(s) (*p*-value 0.58) (Fig. 1c). As 5ALA positive infiltrative margin GBM represents a sub-population(s) in closest proximity to residual disease spared by surgery, this finding justifies a rationale to repurpose OLA for localized interstitial administration.

### High-grade glioma sensitivity to olaparib and radiation in vitro

For in vitro molecular analyzes, we focused on 9 L cells to permit direct comparison with 9 L allografts intended for in vivo efficacy evaluation, and one GBM cell line (U251) as an exemplar. 9 L cells were first assessed in vitro upon exposure to OLA, radiotherapy (XRT), or a combination of OLA and XRT, to enable decoupling of radiosensitization and anti-proliferative effects conferred by OLA. Although OLA (3 µM) alone induced a marked impairment of 9 L clonogenic growth after 7 days, a combination of OLA and 3 Gy XRT induced a significant reduction of clonogenic growth (mean number of colonies, 91.6 ± 10.9), relative to OLA alone (127.6 ± 8.3), XRT alone (170.3 ± 9.4), or untreated control (265.6 ± 5.6) (*p* < 0.05, *p* < 0.001, *p* < 0.001, respectively; Fig. 1d).

### Olaparib confers high-grade glioma sensitivity to DNA damage in vitro

To elucidate whether OLA sensitizes high-grade glioma cells to radiation alone, or more broadly to DNA damaging insults (TMZ or ETOP), flow cytometry was used to quantify proportions of early (AnnexinV + ) and late (AnnexinV+ Propidium Iodide (PI) +) apoptotic/necrotic cells. The total counts of single-positive (AnnexinV^+^PI^-^) and double-positive (AnnexinV^+^PI^+^) cells were measured. In all cell lines tested, distinct populations of AnnexinV^+^PI^-^ and AnnexinV^+^PI^+^ cells were observed following 72 hours of treatment. For both U251 GBM and 9 L rat gliosarcoma cells, the mean proportion of early apoptotic cells in OLA/XRT/TMZ (U251: 45.5% ± 1.0; 9 L: 29.6 ± 1.2) and OLA/XRT/ETOP (U251: 35.93% ± 0.6; 9 L: 7.4% ± 0.5) combinations, was significantly greater relative to OLA/XRT (U251: 19.5% ± 1.20; 9 L: 2.3% ± 1.2) (Fig. 1e, f). For both U251 and 9 L cells, the proportion of late apoptotic cells in OLA/XRT/TMZ (U251: 31.6% ± 2.3; 9 L: 9.8% ± 0.4) was significantly greater relative to OLA/XRT (U251: 20.4% ± 4.2; 9 L: 3.9% ± 0.4). Whilst the proportion of late apoptotic cells in OLA/XRT/ETOP (6.7% ± 0.5) was significantly greater than in OLA/ETOP (3.3% ± 0.3) alone in 9 L cells, there was no significant difference observed in U251 cells (Fig. 1g, h).

### Olaparib induces cell cycle alterations in high-grade glioma in vitro

**Fig. 2:**
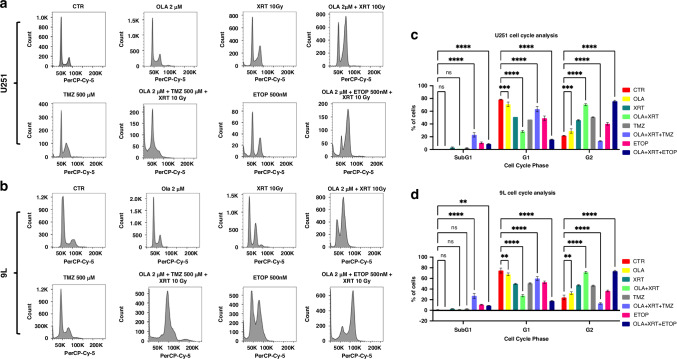
Cell cycle alterations in glioma cells induced by olaparib exposure in vitro. U251 and 9 L high-grade glioma cells were exposed to OLA as single agent, combined with XRT, or combined with XRT and DNA damaging agents TMZ or ETOP. Cells were treated with drug compounds 24 h post-seeding, XRT administered 48 h post-seeding, and cell cycle analyzes conducted 72 h post-seeding. OLA alone, or in combination with XRT, XRT/TMZ or XRT/ETOP, significantly decreased the proportion of G1 cells for U251 (**a**, **c**) and 9 L treatment (**b**, **d**) relative to vehicle-only control cells (CTR). The G2/M peak was significantly decreased in both U251 and 9 L cells upon OLA/XRT/TMZ exposure and increased upon either OLA/XRT or OLA/XRT/ETOP exposure relative to control cells. A significant increase in the sub-G_0_/G_1_ fraction was detected only after OLA/XRT/TMZ or OLA/XRT/ETOP treatment for both U251 and 9 L cells (**c**, **d**). ****p < 0.0001; ***p = 0.0001, **p = 0.001.

We further assessed cell-cycle changes following OLA and combination treatments using flow cytometry with PI staining in 9 L and U251 cells. Cells were treated with OLA and either TMZ or ETOP, radiated after 24 h and sampled after 72 h. OLA (2 μM), either alone, or in combination with XRT, XRT/TMZ or XRT/ETOP, significantly decreased the proportion of cells in G1 phase in both U251 (Fig. 2a, c) and 9 L (Figs. 2b, d) cells. This was concomitant with an increase in proportion of cells in G2/M phase treated with OLA alone, in combination with XRT and in combination with XRT/ETOP in both U251 (Fig. 2a, c) and 9 L (Figs. 2b, d) cells, but not in combination with XRT/TMZ. Of note, a substantial increase in the sub-G1 fraction was detected only in cells treated with a combination of OLA/XRT/TMZ and OLA/XRT/ETOP (Fig. 2c, d). These results confirmed that a decrease in metabolic viability and impairment of clonogenic growth observed in 9 L and U251 cells may be partly mediated by cell-cycle perturbations, whereby OLA-mediated combined DNA damage- and radio-sensitization is more pronounced than OLA-mediated radiosensitization alone.

### Olaparib in combination with radiation and TMZ-mediated genotoxic insult induces DNA damage in high-grade glioma in vitro

**Fig. 3:**
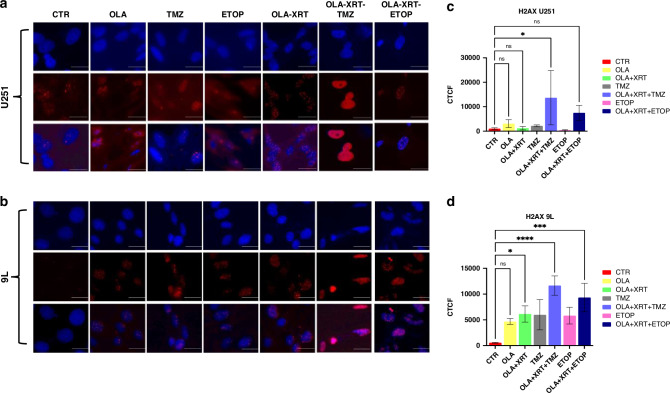
Induction of γH2AX-induced DNA damage upon olaparib exposure. U251 and 9 L cells were exposed to OLA, TMZ or ETOP as single agents, OLA combined with XRT, or OLA combined with XRT and TMZ or ETOP. Cells were treated with drug compounds 24 h post-seeding, XRT administered 48 h post-seeding, and γH2AX immunofluorescence analyzes conducted 72 h post-seeding (**a**, **b**). A significantly higher number of nuclear γH2AX-positive foci was observed after combined OLA/XRT/TMZ in both U251 and 9 L cells relative to OLA/XRT (*p = 0.01). A marked, but non-significant increase in γH2AX-positive foci was observed after combined OLA/XRT/ETOP treatment in U251 and 9 L cells, relative to OLA/XRT (**c**, **d**). *CTCF, corrected total cell fluorescence; Blue – DAPI, Red – γH2AX; CTR, vehicle-only control*. *Primary antibody – anti-phospho-histone H2A.X (Ser139) 1:500 (Merk-Millipore), secondary antibody conjugated to Alexa fluor 594, absorbance/emission 590/617; counterstain – Hoechst 33342 absorbance/emission 345/478; scale bar, 100μm*.

To determine if OLA sensitizes glioma cells to DNA damage, we measured γH2AX phosphorylation in nuclear foci as a proxy for DNA double strand breaks [36] via immunofluorescence in cells treated with OLA and combination treatments as per cell cycle analyzes. Elevated γH2AX expression was observed upon combined OLA/XRT/TMZ 4-days post-treatment in both U251 and 9 L cells, relative to OLA/XRT (Fig. 3a, b). OLA/XRT/TMZ treatment conferred a significant increase in γH2AX expression relative to OLA/XRT in both in vitro models (Fig. 3c, d), whereas OLA/XRT/ETOP treatment conferred a marked, but non-significant increase in U251 and 9 L cells, relative to OLA/XRT (Fig. 3c, d). Protein blots detected elevated phospho-γH2AX expression in 9 L cells treated with OLA alone or OLA/XRT, consistent with immunofluorescence data (Fig. 3d) (relative to XRT alone and vehicle-only control), and cleaved PARP-1 only in cells treated with OLA alone or OLA/XRT (Fig. S1).

### Olaparib potentiates DNA damaging interventions to confer a survival benefit in vivo

**Fig. 4:**
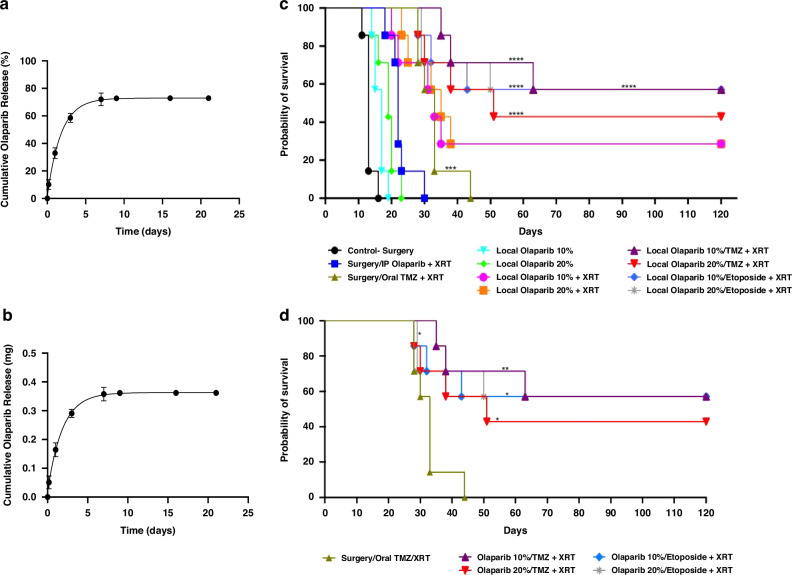
In vivo efficacy of PLGA/PEG-mediated interstitial delivery of olaparib as mono or combination therapy in orthotopic malignant glioma allografts. Exponential plateau fitted in vitro cumulative % (**a**) and amount (mg) (**b**) release of OLA from PLGA/PEG matrices loaded with 500 mg of drug. The release study was performed in PBS (pH = 7.4) at 37 °C and OLA quantified by HPLC over a 21-day period. Error bars indicate the standard deviation from six independent matrices. **c** Kaplan–Meier overall survival plots of randomized F344 rats implanted with 9 L allografts and treated 5-days post allograft implant as follows (*n* = 7 per arm): (*Control arms*) surgery alone; surgery + intraperitoneal (IP) OLA (50 mg/kg daily for 5 days) + XRT; surgery + XRT + oral TMZ by gavage (50 mg/kg daily for 5 days) (clinical standard-of-care); (*Treatment arms*) surgery + PLGA/PEG loaded with 10% *w/w* OLA; surgery + PLGA/PEG loaded with 20% *w/w* OLA; surgery + PLGA/PEG loaded with 10% *w/w* OLA + XRT; surgery + PLGA/PEG loaded with 20% *w/w* OLA + XRT; surgery + PLGA/PEG loaded with 10% *w/w* OLA / 20% *w/w* TMZ + XRT; surgery + PLGA/PEG loaded with 20% *w/w* OLA / 20% *w/w* TMZ + XRT; surgery + PLGA/PEG loaded with 10% *w/w* OLA / 50% *w/w* ETOP + XRT; surgery + PLGA/PEG loaded with 20% *w/w* OLA / 50% *w/w* ETOP + XRT. **d** Depiction of comparisons only between post-surgical combination treatment groups followed by XRT, relative to clinical standard chemotherapy and XRT, demonstrating a significant long-term survival benefit. XRT administered at 10 Gy, 5 days after polymer/drug implant. Animals still alive after 120 days post-surgery and polymer implant were designated LTS. (*p < 0.05; **p < 0.01; ***p < 0.001; ****p < 0.0001).

To first confirm that PLGA/PEG is a suitable drug delivery system capable of administering OLA, we showed that PLGA/PEG microparticles which were mixed with 500 mg OLA and a PBS carrier (1.0: 0.8 (*w*/*v*) polymer: PBS), retained sintering ability at 37 °C to form polymeric matrices. This demonstrates that the OLA-loaded PLGA/PEG formulation can self-assemble at body temperature. In vitro, 33% of loaded OLA was released from PLGA/PEG matrices on day 1, 60% cumulatively released by day 3, continuing to 73% released by day 16 (Fig. 4a, b). High performance liquid chromatography (HPLC) chromatograms show no variance in the retention time of OLA standards and OLA in release media, indicating stability of OLA in PLGA/PEG matrices (Fig. S2).

To assess safety and efficacy of intra-cavity delivery of OLA, we utilized 9 L orthotopic allografts, an aggressive immunocompetent model previously the basis of preclinical development of Gliadel^®^ wafers[37, 38]. Orthotopic tumors (2 mm^3^ allografts) were first surgically resected to create a tumor cavity. PLGA/PEG paste loaded with OLA was applied to the post-surgical tumor cavity (5 days post 9 L allograft implantation) ensuring close apposition to the cavity lining. Seven animals per treatment arm and inclusion of concomitant XRT and oral TMZ as an IDH-WT GBM standard chemotherapy and radiation control arm, ensured statistical significance within a clinically relevant powered therapy study. Long-term survivors (LTS) were designated as those animals still alive at Day 120 post-treatment, as previously reported by us [39, 40].

No significant difference in overall survival was observed between animals treated with surgery alone and animals treated with either surgery followed by systemic OLA and XRT, or post-surgical localized delivery of OLA alone at either 10% or 20% *w/w* PLGA/PEG: OLA. Only the GBM clinical Stupp protocol conferred a significant survival advantage relative to surgery alone (*p* < 0.001), albeit with no LTS (Fig. 4c). In marked contrast, animals treated with post-surgical localized delivery of OLA at either drug dose concomitant with XRT, conferred a significant survival advantage relative to surgery alone, with 2/7 (28%) representing LTS in both treatment arms (*p* < 0.0001 for all comparisons).

We next determined whether delivery of DNA damage agents TMZ or ETOP (at doses previously shown by us to be efficacious) significantly enhanced OLA-mediated XRT sensitization. Data indicated that survival was further potentiated by combining localized post-surgical combined delivery of OLA/TMZ concomitant with XRT, with 4/7 (57%) and 3/7 (43%) LTS for 10% or 20% *w/w* PLGA/PEG: OLA, respectively. Similarly, post-surgical combined OLA/ETOP concomitant with o XRT conferred significant efficacy with 4/7 (57%) and 5/7 (71%) LTS for 10% or 20% *w/w* PLGA/PEG: OLA respectively (Fig. 4c). Data is shown separately to highlight comparison between post-surgical OLA/TMZ or OLA/ETOP concomitant with XRT, relative to clinical Stupp protocol (Fig. 4d). OLA/TMZ or OLA/ETOP combinations in the absence of concomitant XRT was not assessed, as standard clinical treatment includes XRT.

**Fig. 5:**
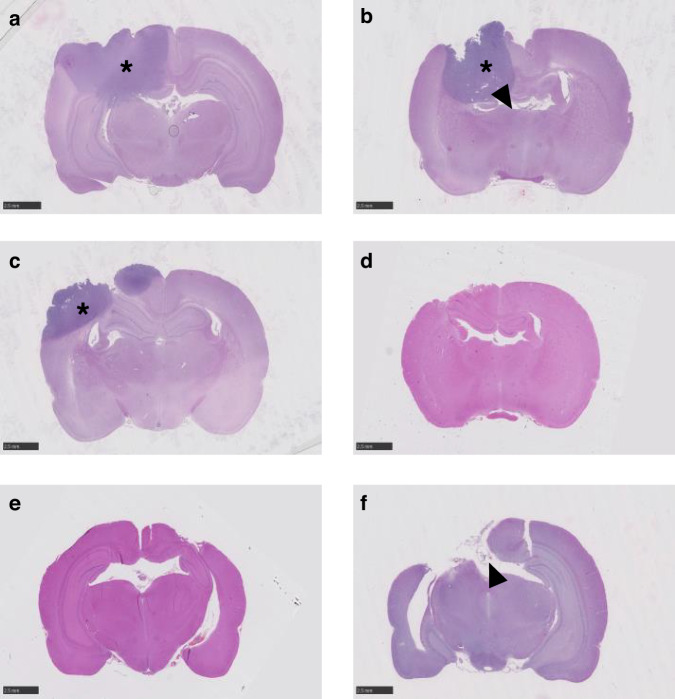
Whole-brain histological confirmation of efficacy upon PLGA/PEG-mediated interstitial delivery of olaparib to orthotopic 9 L gliosarcomas. Animals treated with (**a**) Surgery/intraperitoneal OLA/XRT (Day 22), (**b**) surgery/oral TMZ/XRT (Day 33) and (**c**) surgery/ OLA 20% *w/w* (Day 15), show recurrent tumor extent and cellular dense regions (denoted by *) within the tumor resection cavity (delineated by arrowhead) with visible infiltration of adjacent brain parenchyma. Animals treated with (**d**) surgery/OLA 20% *w/w*/XRT (Day 120), (**e**) surgery/OLA 20% *w/w*/TMZ 20% *w/w*/XRT (Day 120) and (**f**) surgery/OLA 20% *w/w*/ETOP 50% *w/w*/XRT (Day 15), show glial scar formation but with no visible recurrent tumor cells within the surgical resection site (denoted by arrowhead) and brain parenchyma beyond. All images were taken at x40. Scale bar **a**–**h** = 2.5 mm. ‘Days’ = number of days post-polymer implant; oral TMZ administered at 50 mg/kg/day for 5 days (Days 5–9); radiotherapy (XRT) administered as an external beam single dose of 10 Gy directly post-surgery.

To confirm that the observed survival bsenefit in PLGA/PEG/OLA concomitant with XRT or DNA damaging agent treatment groups was directly attributable to efficacious interstitial delivery, histological assessment of surgical resection margins and neighboring parenchyma were conducted on rat brains post-sacrifice. Whole-brain histological analyzes ensured that any distal tumor recurrence within the contra-lateral hemisphere could be detected. Animals treated either with surgery alone (Day 16) (Fig. S3A), surgery followed by systemic (intraperitoneal) delivery of OLA concomitant with XRT (thus mimicking current phase II OLA clinical trials) (Day 22), treated with GBM standard treatment (surgery, oral TMZ and concomitant XRT) (Day 33), or treated with post-surgical delivery of PLGA/PEG/OLA (10–20% *w*/*w*) (Day 15), showed extensive tumor recurrence and dense cellularity within and beyond the surgical cavity (Fig. S3B and Fig. 5a–c). Of note, an animal treated with post-surgical delivery of PLGA/PEG/OLA (10% *w*/*w*) concomitant with XRT (Day 22) which required euthanasia due to adverse effects, was predictably associated with extensive tumor recurrence (Fig. S3C). In marked contrast, animals identified as LTS, treated with post-surgical delivery of PLGA/PEG/OLA (10-20% *w*/*w*) concomitant with XRT (Day 22 and Day 120) (Fig. S3D and Fig. 5d), showed no visible recurrent tumor within the surgical resection site and brain parenchyma beyond. Histological evidence of efficacy for LTS animals was also confirmed for post-surgical delivery of PLGA/PEG/OLA/TMZ (10–20% *w*/*w* for OLA; 20% *w*/*w* for TMZ) concomitant with XRT (Day 120) (Fig. S3E and Fig. 5e), and PLGA/PEG/OLA/ETOP (20% *w*/*w* and 50% *w*/*w* respectively) (Day 15) (Fig. 5f), with brains showing no visible recurrent tumor within the surgical resection site, adjacent brain parenchyma, or in the contralateral hemisphere.

## Discussion

Standard post-surgical administration of TMZ monotherapy with concomitant XRT is moderately effective as DNA damaging therapy for IDH-WT GBM ( ~ 2-month survival benefit) [2, 41] over XRT alone, but in most cases does not result in a durable response. Preclinical/clinical development and translation of inhibitors to potentiate this DNA damage is therefore a crucial unmet clinical need. The protein family of PARPs constitute multidomain proteins which are associated with critical cellular homeostatic functions (e.g., DNA damage repair, protein aggregation, endoplasmic reticulum stress) [13]. Tumor cells which are incapable of efficient double strand repair are particularly sensitive to PARP inhibitor-associated prevention of single DNA strand breaks repair, which destabilizes DNA, resulting in double strand breaks [42, 43]. As such, there has been considerable interest in PARP inhibition by OLA as a treatment strategy for solid tumors [15–20, 42–44] including IDH-WT GBM [21–25, 45, 46]. Systemically delivered oral OLA has been shown clinically to be well tolerated in newly diagnosed and relapsed GBM with detectable drug penetration in tumor core and margin [21, 24], and systemic OLA delivery recently shown to be enhanced using focused ultrasound in a diffuse midline glioma model [27]. However, there have been no studies to assess localized, interstitial OLA delivery for high-grade glioma (nor any solid tumor).

The lack of sufficient objective response from molecular targeted therapeutics at phase III clinical trials predicated on IDH-WT GBM genomic data [47–51], is in considerable part due to a neglect of intra-tumor heterogeneity [52–57]. Our RNAseq data on the GBM infiltrative margin based on 5ALA fluorescence-based isolation, offers a clinical validation of the OLA drug target (i.e., PARP-1) at the outset for preclinical drug delivery efficacy assessment, as PARP-1 mRNA expression was detected in infiltrative GBM cells at comparable levels to intra-tumor regions. It is therefore reasonable to suggest that infiltrative disease remaining post-surgery may also express similar PARP-1 levels. Our overall findings support the hypothesis that localized, post-surgical OLA delivery, mediates both radio- and DNA damage-sensitization. Furthermore, OLA-mediated combined sensitization to radiation and genotoxic agents confers greater efficacy than radiation alone. That OLA alone impairs clonogenic growth of high-grade glioma cells in vitro but is inferior to standard treatment when locally administered in vivo, is likely due to intracellular uptake of high drug concentration by tumor cultures, relative to drug concentration which reaches infiltrative disease in vivo. This interpretation is supported by in vitro uptake and associated cytotoxicity in triple-negative breast cancer and cervical cancer cells upon exposure to OLA alone [43, 58].

Our observation of increased sub-G1 fractions is consistent with a hypothesis stating that by inhibiting repair of single‐strand DNA breaks, OLA enhances the lethality of genotoxic treatments such as XRT, ETOP and TMZ. As a combination of OLA/XRT results in G1/S decrease and G2/M increase (consistent with mitotic cell death in response to unrepaired double strand breaks) but is not associated with apoptosis as determined by a sub-G1 fraction, this indicates that OLA-mediated DNA damage agent sensitization is more marked than radiosensitization. In support of this notion, a significant increase in phosphorylated γH2AX (evidence of unrepaired DNA damage) was observed in high-grade glioma cells treated with OLA/XRT/TMZ relative to OLA/XRT. This has implications for concomitant therapy whereby GBM patients receiving multimodal standard Stupp protocol [2] may be more likely to be responsive.

**Fig. 6:**
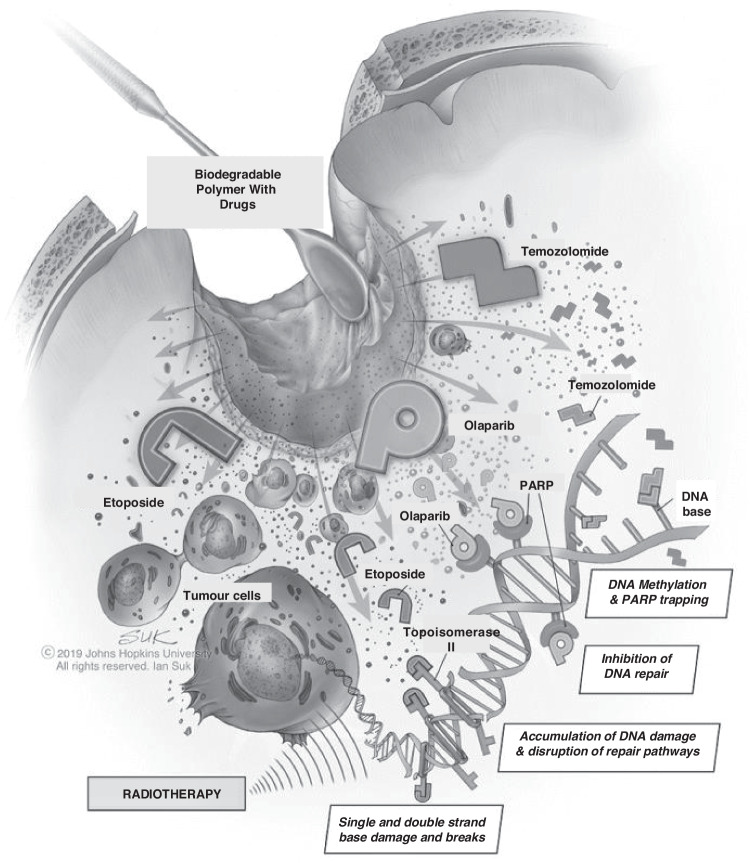
Schematic illustration of combination chemotherapy with OLA, ETOP and TMZ, with concomitant radiotherapy, in post-resection high-grade glioma. The resection cavity is filled with drug-impregnated biodegradable polymeric paste, with subsequent diffusion of OLA, ETOP and TMZ towards the tumor margins and infiltrating cells. DNA damage through multiple putative mechanisms is achieved in combination with radiotherapy.

Overall survival and LTS upon interstitial polymeric delivery of BCNU/carmustine in a 9 L preclinical model was previously reported as accurately predictive of efficacy in a clinical trial for Gliadel^®^, thus validating 9 L as a testbed for realistic clinical translation [10, 37, 59, 60]. Although interpretation is confined to one allograft model, our observation of no survival benefit conferred by post-surgical systemic OLA delivery concomitant with XRT in 9L-bearing animals, relative to surgery alone treatment, cautions against a supposition that current phase II/III OLA trials for GBM [25, 45] will yield an objective clinical response. Similarly, polymeric interstitial delivery of OLA post-surgery was inferior to GBM standard therapy, indicating insufficient OLA concentrations to confer single-agent anti-cancer effects, as observed in metabolic assays using 9 L cells in vitro. In contrast, overall survival benefit and an associated LTS of 28% conferred by interstitial OLA delivery in combination with XRT (relative to surgery alone), demonstrates the localized OLA concentrations were sufficient to induce radiosensitization. Moreover, as this effect was potentiated by combined OLA/TMZ or OLA/ETOP concomitant with XRT (as determined by LTS of 43–71%), our findings indicate that DNA damaging agents significantly enhance localized OLA-mediated XRT sensitization, the precise mechanisms for which remain unclear in this experimental context (Fig. 6) but are likely to involve the accumulation of unrepaired DNA damage. We note that interstitial delivery of OLA was comparably efficacious at 10% or 20% dose when combined with either TMZ or ETOP, suggesting that 10% OLA is the maximum XRT sensitization dose in this context, DNA damage effect induced by TMZ and ETOP is comparable and that DNA damage induced by XRT is the limiting factor.

Whole brain histological evidence shows that long-term surviving animals were associated with disease-free brains. This raises an overarching caveat. Current rodent allograft and xenograft orthotopic models are likely sub-optimal for recapitulating the true GBM infiltrative extent which is consistently observed clinically. Genetically engineered rodent models which better recapitulate infiltrative GBM, may offer a preclinical testbed predictive of phase II/III objective response in GBM clinical trials. Nevertheless, Gliadel^®^ remains an exemplar of efficacious interstitial delivery, with a median survival increase of ~2 months relative to placebo-treated patients in an initial clinical trial [8], with recent reporting of 3-year ( ~ 40% of patients) and 2-year ( ~ 30% of patients) overall survival [9, 60]. It is important to note that no molecular targeted therapeutic predicated on patient-tailored genomic data has demonstrated efficacy in phase III clinical trials for GBM. Since 9 L allograft tumors recur rapidly post-standard therapy (surgery, TMZ and XRT), we may infer from our experimental data, that localized delivery of OLA ± TMZ/ETOP concomitant with radiation, is sufficient to efficaciously target residual infiltrative disease. Furthermore, our observed LTS is comparable to LTS for both localized BCNU/carmustine in 9 L allografts (which formed the preclinical basis for the clinical translation of Gliadel^®^), and to LTS of BCNU/TMZ-treated 9 L allografts [38, 61]. As most IDH-WT GBM patients (excluding a minority of patients who will receive biopsy surgery only) will undergo resective surgery as initial intervention, localized drug delivery will remain a viable option to initiate oncological treatment post-surgery. The survival benefit to Gliadel^®^-administered GBM patients encourages consideration of PLGA/PEG polymeric delivery of OLA. For clinical trial readiness, our PLGA/PEG/OLA formulation requires manufacturing of a prototype suitable for application to a human GBM resection cavity, for which a dual-syringe system is envisaged.

## Supplementary information

Supplementary Files

## Data Availability

Raw RNA-seq data for spatially distinct unsorted GBM regions and 5ALA fluorescence activated cell sorted cells have been deposited at the ArrayExpress with accession number: EMTAB-8743. All other data are available in the main text or supplementary materials.
