# Supplementary material for: Neurosurgical application of olaparib from a thermo-responsive paste potentiates DNA damage to prolong survival in malignant glioma
Source: Br J Cancer. 2024 Oct 22;131(11):1858–68. doi: 10.1038/s41416-024-02878-2 (PMC11589713; doi:10.1038/s41416-024-02878-2)
Supplement: Supplementary file 1 — Supplementary Files [file 41416_2024_2878_MOESM1_ESM.docx]

Materials and Methods

**Western Blotting**

9L cells were treated with Olaparib 2uM, XRT 5Gy and combination Olaparib-XRT, and pellets were harvested and frozen. Protein concentration was then determined with BCA Assay (GE Healthcare, Marlborough, MA, USA) and membranes blotted with anti-AIF (1:1 000; Cell Signaling), anti-PARP (1:1 000; Cell Signaling), and anti-phospho-Histone H2A.X (Ser139) (1:500, Merk-Millipore, Burlington, MA, USA). Membranes were incubated with horseradish peroxidase-conjugated secondary antibody (1:5 000; Jackson Laboratory, Bar Harbor, ME, USA), and band density was normalized to the GADPH control signal.

Figures & Tables


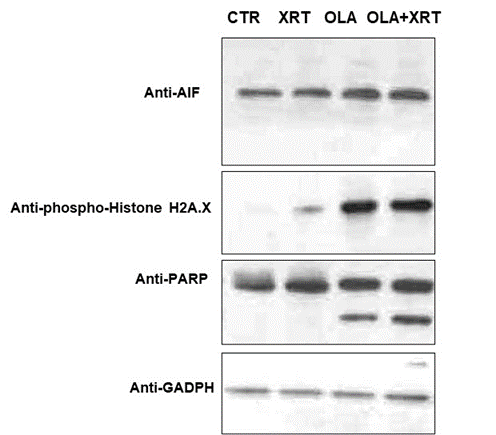


**Fig. S1. OLA induced DNA damage in a PARP pathway specific manner is potentiated by radiation therapy in 9L gliosarcoma cells.** Western Blot shows a uniform increase in the cleaved-PARP fraction and a reduction in total PARP after 48 hours of treatment with OLA and a combination of OLA + XRT. Similarly, the levels of AIF protein, a caspase-independent effector that regulates mitochondrial permeability and initiates nuclear DNA condensation, and is released after PARP cleavage into PAR, were elevated upon 9L exposure to OLA and a combination of OLA + XRT, relative to vehicle-only control and treatment with XRT alone. Relatively higher expression of H2A.X, a marker of DNA damage, was also observed after OLA and a combination of OLA + XRT.


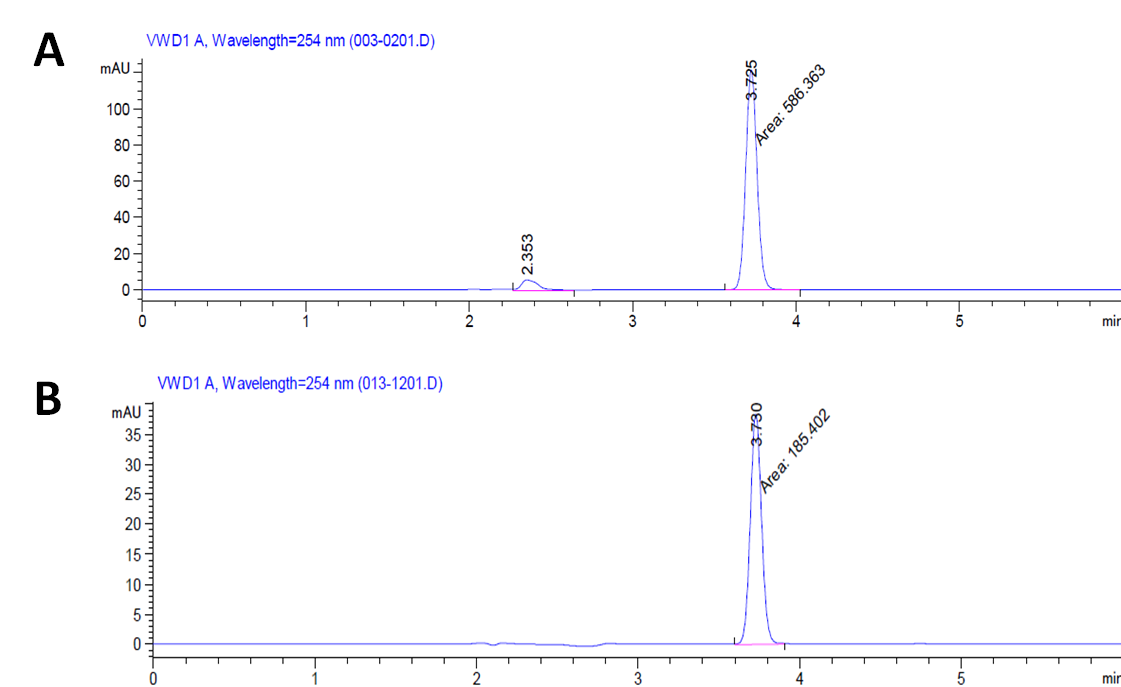


**Fig. S2. Representative HPLC chromatograms for the analytical detection of OLA**. (A) OLA standard (25 µg/mL), showing peak of interest at a retention time of *c.* 3.3 minutes. (B) Release media containing OLA following 4 hours of incubation at 37 °C, showing conservation of retention.


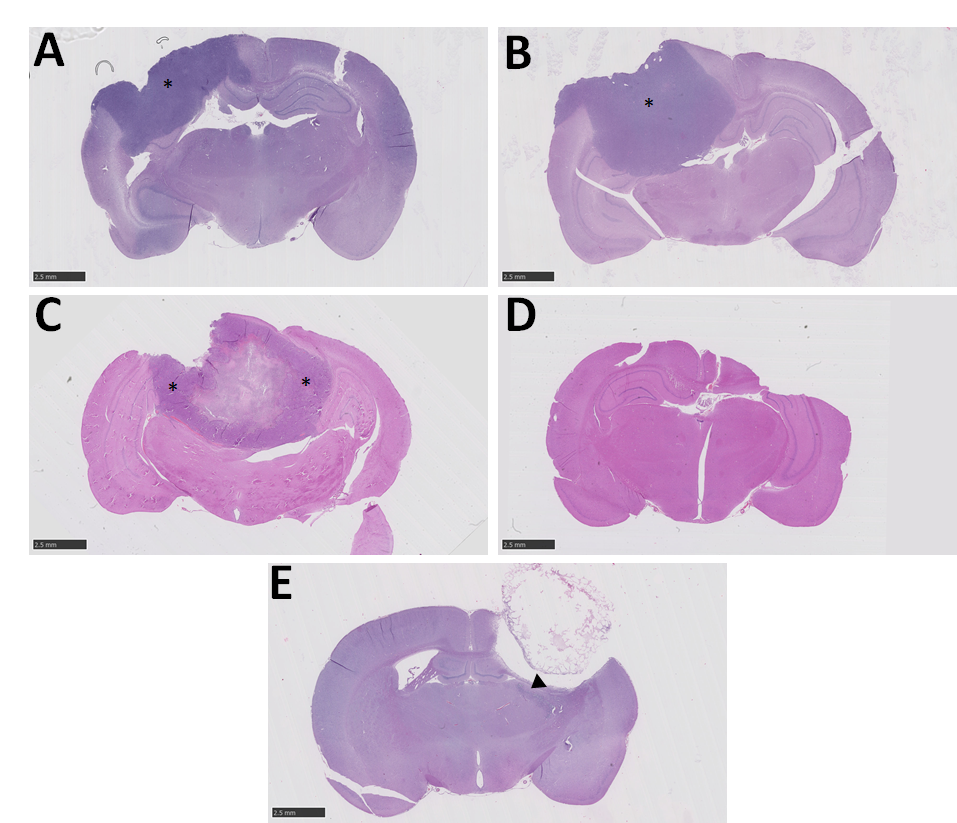


**Fig. S3. Whole-brain histological confirmation of efficacy after post-PLGA/PEG-mediated interstitial delivery of OLA to orthotopic 9L gliosarcomas**. *Hematoxylin and eosin staining*: Animals treated with (A) Surgery alone (Day 16), (B) surgery / OLA 10% *w/w* (Day 15) and (C) surgery / OLA 10% *w/w* / XRT (Day 22), show extensive tumor recurrence and dense cellularity (denoted by *) within the surgical resection cavity, with tumor cells visibly infiltrating brain parenchyma. In marked contrast, animals treated with (D) surgery / OLA 10% *w/w* / XRT (Day 120) and (E) surgery / OLA 10% *w/w* / TMZ 20% *w/w* / XRT (Day 28), show gliotic scarring but with no visible recurrent tumor cells within and the surgical resection site (denoted by arrowhead) and brain parenchyma beyond. *All images taken at x40*. *Scale bar A-H 500µm*. *‘Days’ = days post-polymer implant; oral TMZ administered at 50 mg/kg/day for 5 days (Days 5-9); radiotherapy (XRT) administered as an external beam single dose of 10Gy immediately after surgery.*
